# Venous Thromboembolism in the Cancer Population: Pathology, Risk, and Prevention

**Published:** 2012-01-01

**Authors:** Susan Hawbaker

**Affiliations:** From CNS Home Hospice and Palliative Care, Carol Stream, Illinois

## Abstract

Patients with cancer have an increased risk of developing venous thromboembolism (VTE) and the incidence of these events has been increasing over the past decade. Venous thromboembolic events include both deep venous thrombosis and pulmonary embolism. These events contribute to higher morbidity and mortality rates. Understanding the complex pathogenesis of and risk factors for cancer-associated VTE will help guide advanced practitioners to improve outcomes with prophylaxis. The American Society of Clinical Oncology, the National Comprehensive Cancer Network, and the European Society of Medical Oncology have utilized this information and developed evidence-based guidelines for prophylactic management for those who are at highest risk of developing cancer-associated VTE. This review will discuss the impact of cancer-associated VTE as well as its underlying pathogenesis, risk factors, and current recommendations for prophylaxis.

Patients with cancer have a two- to sixfold increase in the risk of developing venous thromboembolism (VTEs; Samama, Dahl, Quinlan, Mismetti, & Rosencher, 2003), and the incidence continues to increase with cancer-associated VTE representing nearly 20% of all cases (Heit et al., 2002). These cases have shown to have a major impact on prognosis, mortality, and morbidity of the cancer population. Not only does the incidence of VTE impact prognosis, but it also deters and complicates treatment options. This increased awareness of the impact of VTE on mortality and morbidity in cancer has led to a need to understand the underlying pathogenesis, risk factors, and possible prophylactic options available.

Armand Trousseau first identified a relationship between cancer and coagulopathy in 1865 (Caine, Stonelake, Lip, & Kehoe, 2002). Due to the increasing incidence and impact that VTE has on cancer, researchers continue to expand on Trousseau’s discovery by exploring the complicated interactions between host cells, cancer cells, and treatment regimens that underlie the pathogenesis of coagulopathy in malignancy.

The purpose of this review is to discuss the epidemiology of VTE and malignancy, explore the known hypercoagulable pathogenesis associated with cancer VTE, understand the risk factors for cancer-associated VTE, and outline the current recommendations and guidelines for prevention of VTE in the oncology setting.

## Epidemiology

The incidence of VTE in the cancer population has been increasing over the past decade; identifying the epidemiology of VTE in this population will help oncology advanced practitioners (APs) understand its prognosis, prophylaxis, and treatment. Between 1980 and 2000 the incidence rates of VTE increased from 0.6% (Levitan et al., 1999) to 4% (Khorana, Francis, Culakova, Kuderer, & Lyman, 2007a; Stein et al., 2006), which represents nearly a 400% increase. To determine possible causes for this increase, studies have examined the relationship among VTE incidence, cancer-related therapies, and diagnostic procedural usage.

In the setting of cancer-related therapies, patients receiving chemotherapy saw nearly a 50% increase in their incidence rates of VTE within the past decade (3.9% to 5.7%), whereas cancer patients undergoing surgery experienced no change (Khorana et al., 2007a). It has been speculated that the increase in the use of diagnostics has also had an impact on the overall increasing rate of cancer-associated VTE. However, studies have found that the use of newer chemotherapeutic regimens as well as the use of high-resolution computed tomography (CT) is likely the cause (Khorana et al., 2007a). The newer diagnostic technology, such as high-resolution CT, provides better visualization, leading to an increase in VTE findings. However, it has been estimated that the actual incidence rate of VTE in the oncology population is underestimated, as postmortem studies have found VTE in nearly 50% of all cancer patients (Goldenberg, Kahn, & Solymoss, 2003).

Specific factors associated with an increase in incidence of VTE include time from diagnosis, aggressiveness of cancer, and metastatic involvement. The relative risk of developing VTE is seven times higher in patients who have active cancer, with the highest incidence rate occurring within the first few months after diagnosis (Wun & White, 2009). For example, the incidence rate in colon cancer is 5% in the first 6 months after diagnosis vs. 1.4% in the following 6 months (Wun & White, 2008). In addition, advanced cancers are associated with a two to three times higher incidence of fatal VTE (Rodrigues, Ferrarotto, Filho, Novis, & Hoff, 2010). Patients who have quick metastatic spread compared to a large extent of metastasis have a higher occurrence rate of VTE (Wun & White, 2009; Rodriquez et al., 2007).

Cancer-associated VTE is a leading cause of morbidity and mortality in both ambulatory and hospitalized patients (Wun & White, 2009; Khorana et al., 2007b). Researchers have identified poor survival and prognosis in cancer patients with VTE compared to cancer patients without VTE, with only 12% surviving past 1 year (Sorensen, Mellemkjaer, Olsen, & Baron, 2000). Both surgery and chemotherapy have had a huge impact on the mortality rates associated with VTE. Patients receiving chemotherapy have a rate of VTE-related death that is 47 times higher than that of the general population (Khorana et al., 2007b). In addition, VTE is the most common cause of death after cancer-related surgery (Agnelli, Bolis, Capussotti, Scarpa, Tonelli, et al., 2006), with a death rate triple that of the noncancer surgical patient (Khosravi-Shai & Perez-Manga, 2009).

## Pathogenesis

**Figure 1:**
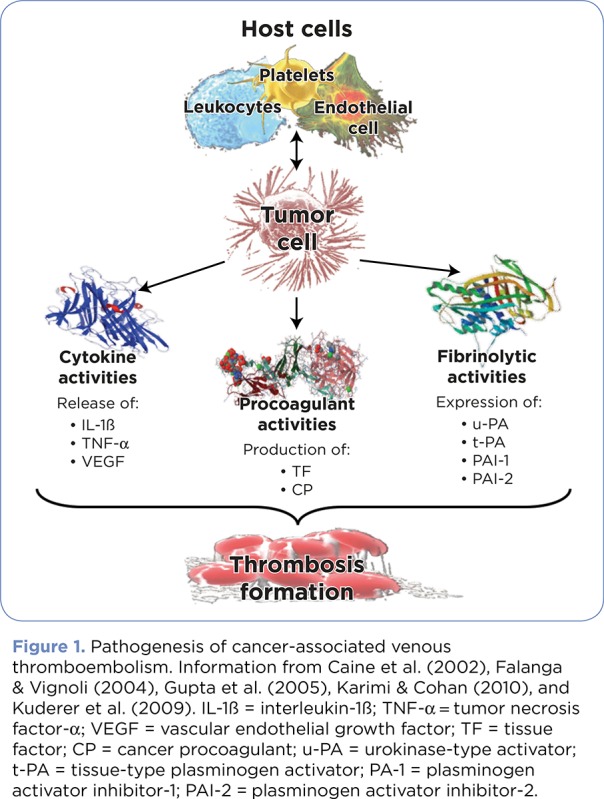
Figure 1. Pathogenesis of cancer-associated venous thromboembolism. Information from Caine et al. (2002), Falanga & Vignoli (2004), Gupta et al. (2005), Karimi & Cohan (2010), and Kuderer et al. (2009). IL-1ß = interleukin-1ß; TNF-α = tumor necrosis factor-α; VEGF = vascular endothelial growth factor; TF = tissue factor; CP = cancer procoagulant; u-PA = urokinase-type activator; t-PA = tissue-type plasminogen activator; PA-1 = plasminogen activator inhibitor-1; PAI-2 = plasminogen activator inhibitor-2.

The pathogenesis of cancer-associated thrombosis is complicated and multifactorial. The basic pathology of thrombosis consists of Virchow’s triad: venous stasis, endothelial damage, and an intrinsic hypercoagulable state. This pathology continues in the oncology patient; however, it is the intrinsic hypercoagulable state that exerts the most influence on this population’s elevated risk (Figure 1).

The tumor cells themselves contribute to the intrinsic nature of clotting in the oncology population. These cells do this with four different mechanisms of action (Table 1): (1) production of procoagulant factors activating thrombosis formation, (2) release of fibrinolytic activities, (3) generation of acute phase reactants, including inflammatory cytokines that activate the clotting cascade, and (4) interaction with host blood cells (Caine et al., 2002; Falanga & Vignoli, 2004; Gupta, Charan, & Kumar, 2005; Karimi & Cohan, 2010).

**Table 1:**
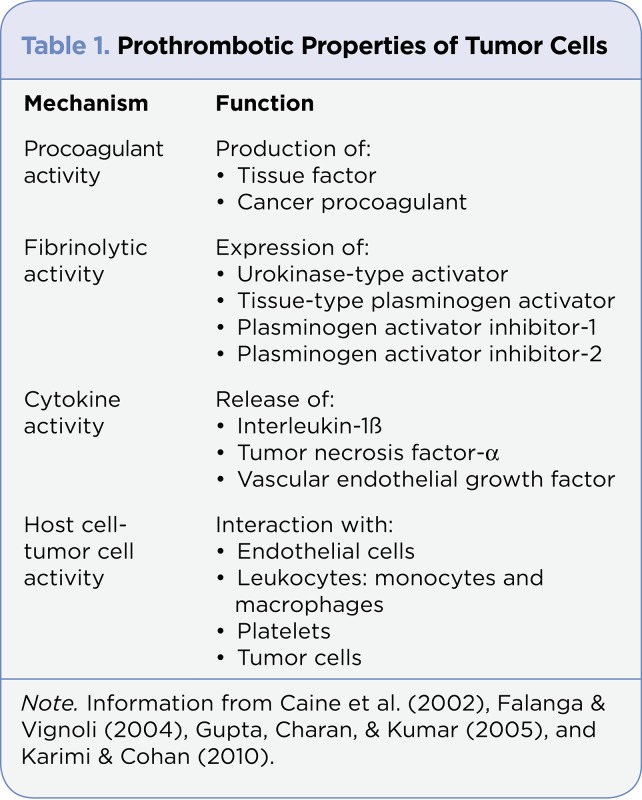
Table 1. Prothrombotic Properties of Tumor Cells

## PROCOAGULANT MECHANISMS

Procoagulant activity refers to when cells promote the formation of fibrin deposits at injured areas (Caine et al., 2002). The main procoagulants expressed in tumor cells are tissue factor and cancer procoagulant (Kuderer, Ortel, & Francis, 2009).

Tissue factor is a protein present on subendothelium, platelets, and leukocytes that forms a complex with factor VII (Kuderer et al., 2009; Rodrigues et al., 2010). This complex then activates factors IX and X in the coagulation pathway (Kuderer et al., 2009). Under normal circumstances, tissue factor is released in response to an inflammatory stimulus; however, in malignant cells tissue factor is expressed in a continuous and increasing manner, which contributes to constant procoagulant activity (Caine et al., 2002; Rodrigues et al., 2010).

Cancer procoagulant, found mainly in malignant tissues, is a protease enzyme that activates factor X of the coagulation pathway directly (Varki, 2007). It is released by many different types of tumor cells, and it actively promotes thrombosis and platelet activation (Karimi & Cohan, 2010).

## FIBRINOLYTIC MECHANISMS

Tumor cells have the capacity to express all the needed inhibitors and activators to regulate the fibrinolytic pathway (Caine et al., 2002). This includes the expression of the activators urokinase-type activator (u-PA) and tissue-type plasminogen activator (t-PA), and the expression of the inhibitors plasminogen activator inhibitor-1 (PAI-1) and plasminogen activator inhibitor-2 (PAI-2) (Caine et al., 2002).

## RELEASE OF CYTOKINES

Tumor cells secrete a large number of different types of proinflammatory cytokines. Release of tumor necrosis factor-á (TNF-á), interleukin-1â (IL-1â), and vascular endothelial growth factor (VEGF) inhibit the normal anticoagulant system by inducing the expression of tissue factor by vascular endothelial cells (VECs) (Caine et al., 2002; Karimi & Cohan, 2010). This downregulates the activation of the protein C system (Goldenberg, Kahn, & Solymoss, 2003), which increases the ability of the vascular wall to attach to leukocytes and platelets, promoting thrombosis formation (Caine et al., 2002; Karimi & Cohan, 2010).

**TUMOR CELL TO HOST CELL INTERACTIONS** 

Malignant cells interact with host cells such as platelets, leukocytes, and endothelial cells to promote thrombosis formation (Kuderer et al., 2009; Karimi & Cohan, 2010). When tumor cells adhere directly to the endothelial wall of the host, the tumor cells release cytokines that contribute to the adhesion of leukocytes and platelets, leading to thrombosis (Caine et al., 2002). Platelet activation is increased when the tumor cells shed and bind to the platelets, allowing platelet aggregation by physical bridging (Caine et al., 2002; Haddad & Greeno, 2006). Tumor cell adhesion to leukocytes, specifically monocytes and macrophages, causes an increase in tissue factor release by these cells and thus an increase in procoagulant activity in host cells (Caine et al., 2002; Gupta et al., 2005).

**ADDITIONAL NONSPECIFIC FACTORS** 

Some chemotherapy agents have been associated with a higher risk of VTE in cancer patients. These agents contribute by increasing the release of procoagulant factors and cytokines due to cellular damage, direct endothelial lesions, and decreased production of the body’s inhibitors to coagulation from hepatotoxicity (Rodrigues et al., 2010). Cisplatin, thalidomide (Thalomid), bevacizumab (Avastin), and lenalidomide (Revlimid) are four agents that have been implicated in cancer- associated VTE. Cisplatin activates platelet aggregation, causes endothelial damage, and increases von Willebrand factor levels (Rodrigues et al., 2010). The antiangiogenic drugs (e.g., thalidomide, lenalidomide, and bevacizumab) increase risk due to the anti-VEGF effects that promote procoagulant activity (Nalluri, Chu, Keresztes, Zhu, & Wu, 2008; Zangari et al., 2009). Thalidomide and lenalidomide alter the normal action of platelets on the endothelium, causing an increase in platelet aggregation and von Willebrand factor (Rodrigues et al., 2010).

Other nonspecific factors contributing to the pathogenesis of thrombosis include immobilization leading to venous stasis, inflammation from necrotic normal or cancer tissues, and foreign body effects such as those of venous access devices (Gupta et al., 2005).

## Risk Factors

Risk factors for cancer-associated VTE can be separated into three categories: patient-, cancer-,and treatment-related factors (Table 2). In addition, new studies are looking into the impact of laboratory biomarkers as predictive tests for risks of VTE in cancer. The interaction between these risk factors is extremely complex, requiring astute assessment to determine VTE threat to each patient. Recently, a risk model for chemotherapy-associated VTE was developed to help assist practitioners to stratify patients into three different risk categories; see Table 3 and discussion below (Khorana, Kuderer, Culakova, Lyman, & Francis, 2008b). The implications of this risk model with current VTE guidelines are still to be determined.

**Table 2:**
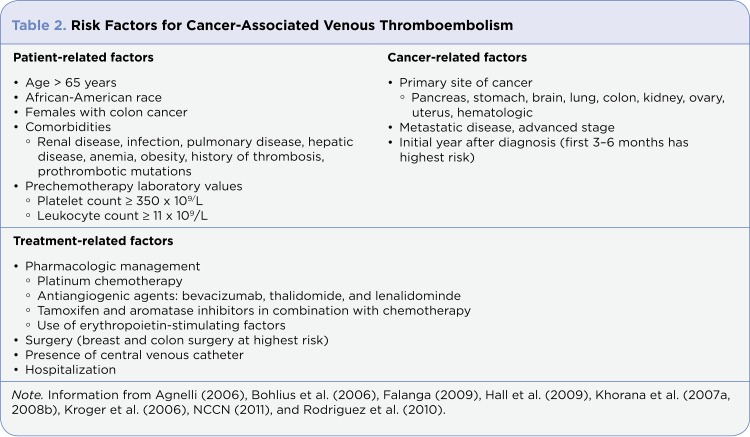
Table 2. Risk Factors for Cancer-Associated Venous Thromboembolism

**Table 3:**
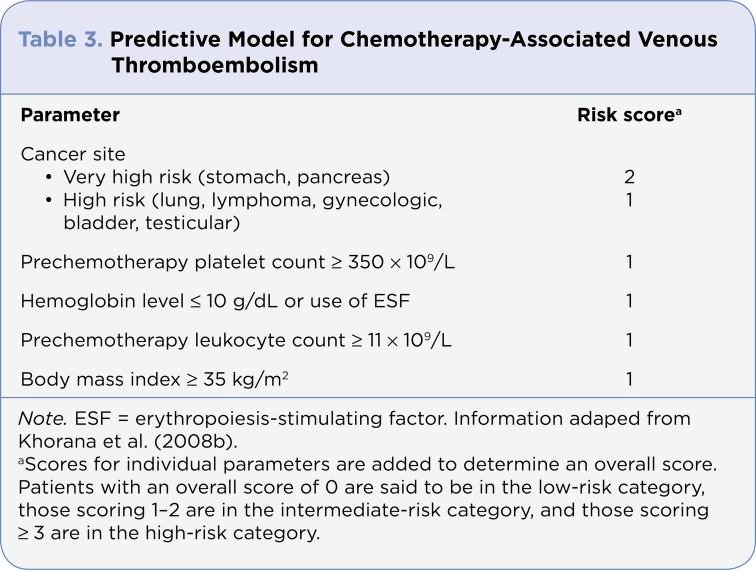
Table 3. Predictive Model for Chemotherapy-Associated Venous Thromboembolism

**PATIENT-RELATED RISK FACTORS** 

Specific patient-related risk factors linked to cancer-associated VTE are age, gender, race, and comorbidities. A study conducted by Khorana et al. (2007a) found that patients over the age of 65 (with a median age of 75) have an elevated risk of VTE (Hall, Andersen, Krumholz, & Gross, 2009). However, a large database study found that patients over 85 years old have a very small degree of protection from cancer-associated VTE for unknown reasons (Hall et al., 2009). The impact that gender has on risk has been controversial. However, studies have implicated an increased risk of VTE with female gender (Khorana et al., 2007a). This is especially true among the colon cancer population (Hall et al., 2009). The black ethnicity has the highest incidence compared to any other ethnicity, with a rate of 5.1% (Khorana et al., 2007a).

Comorbid conditions increasing risk of VTE include renal disease, infection, pulmonary disease, hepatic disease, anemia, obesity, history of thrombosis, and prothrombotic mutations (Khorana et al., 2007a; 2007b.). One large database study found that congestive heart failure, chronic obstructive pulmonary disease, dementia, and atrial fibrillation have a modest protective effect on the risk of VTE in the cancer population (Hall et al., 2009).

**CANCER-RELATED RISK FACTORS** 

Cancer-related risk factors include tumor site, time from diagnosis, and disease stage. Tumor type has an impact on mortality risk. The risk of death from VTE in certain cancers can range from 20% to 40%, whereas other cancers, including breast and prostate, are associated with an insignificant VTE-associated mortality risk (Gross, Galusha, & Krumholz, 2007). Cancer sites associated with the highest incidence of VTE include pancreas, stomach, brain, kidney, uterus, lung, ovary, colon, and hematologic malignancies (Levitan et al., 1999; Khorana et al., 2007a; National Comprehensive Cancer Network [NCCN], 2011). When looking at histology, patients with non–small cell, adenocarcinomas, and mucinous cancers have the highest risk of VTE (Khorana & Connolly, 2009; Falanga, 2009; Varki, 2007). Disease sites associated with the lowest risk of VTE include head and neck, bladder, breast, prostate, esophagus, uterus, and cervix (Levitan et al., 1999; Hall et al., 2009).

Patients with advanced metastatic disease have a higher risk of VTE than those with local-stage cancers (Wun & White, 2009). Researchers identified a twofold increase in risk in cancer patients with distant metastasis compared to those without metastasis (Blom et al., 2006). The underlying etiology of this is unclear; however, it has been implicated that this may be related to poor performance status vs. stage (Khorana & Connolly, 2009). In addition, patients are at greatest risk of VTE within the first year of diagnosis (Sousou & Khorana, 2009). In the second and third years this risk declines rapidly, with a rate of 5.32 in the first year to 0.97 and 0.36 in consecutive years (Hall et al., 2009).

**TREATMENT-RELATED RISK FACTORS** 

Treatment-related risk factors include pharmacologic management, surgery, hospitalization, and the use of central venous catheters (CVCs).

Pharmacologic agents that increase risk of VTE include chemotherapies, hormone therapies, and supportive therapies. Patients receiving chemotherapy have a 2.2-fold increase in risk of VTE compared to patients not receiving chemotherapy (Blom et al., 2006; Khorana et al., 2007a). Treatment with platinum chemotherapies and the newer antiangiogenic agents, such as bevacizumab, thalidomide, and lenalidomide, increases the risk of VTE (Kröger et al., 2006; Khorana et al., 2009). Use of hormone therapy, such as tamoxifen and the aromatase inhibitors, in combination with chemotherapy increases risk of VTE in breast cancer patients (Rodrigues et al., 2010). Cancer patients often rely on erythropoietin-stimulating agents for treatment of chemotherapy-related anemia; however, recent studies have identified that these agents increase the risk of VTE by 67% (Bohlius et al., 2006). In addition, a recent study found that both platelet and blood transfusions contribute independently to an increased risk of VTE in the cancer population (Khorana et al., 2008a).

Surgery is a well-established contributor to VTE risk in the general population; in the oncology population, VTE is the most common cause of death after surgery (Agnelli et al., 2006). Patients who undergo breast and colon cancer–related surgery are at higher risk of VTE compared to other cancer-related procedures (Hall et al., 2009). A recent study identified other risk factors for postoperative VTE in the oncology population, including age greater than 65 years, previous VTE, advanced disease, anesthesia lasting more than 2 hours, and bedrest lasting longer than 3 days (Agnelli et al., 2006). This same study identified that the oncology population has a prolonged risk of thrombosis after surgery that extends past 21 days, thus requiring lengthier anticoagulation protection (Agnelli et al., 2006).

The oncology population relies on the use of long-term indwelling central catheters to provide chemotherapies, stem cell transplantation, or nutrition and hydration support. However, it has been demonstrated that CVCs contribute to an increased risk of VTE complications (Caine et al., 2002; Falanga, 2009). Risk factors for developing VTE after CVC insertion include more than one insertion attempt, ovarian cancer, and previous CVC insertions (Lee et al., 2006).

**BIOMARKERS** 

Several recent studies have identified a relationship between the levels of biomarkers and increasing risk of VTE in cancer. A prospective observational study found that a platelet count greater than 350 × 109/L prior to chemotherapy was indicative of an increased risk of VTE (Khorana, Francis, Culakova, & Lyman, 2005). Another unique indicator of increased risk is a prechemotherapy leukocyte count of more than 11 × 109/L (Khorana et al., 2008b). Other markers that may be associated with increased risk of VTE include elevated tissue factor (Khorana et al., 2007), D- dimers (Sallah et al., 2004), C-reactive protein (Kroger et al., 2006), and soluble P- selectin (Ay et al., 2008).

**RISK MODEL** 

In 2008, Khorana et al. developed and validated a predictive risk assessment tool for chemotherapy-associated VTE. It includes the use of five clinical and laboratory parameters to classify patients into three different risk levels: low, intermediate, and high. These characteristics include cancer site, prechemotherapy platelet count, hemoglobin level, prechemotherapy leukocyte count, and body mass index; see Table 3 (Khorana et al., 2008b). However, additional risk factors may need to be considered, including hospitalization, stage of cancer, thrombogenicity of chemotherapy, and patient comorbidities (Falanga, 2009).

## Review of Current Guidelines for VTE Prophylaxis in Cancer

In response to the overwhelming impact that cancer-associated VTE has had, several specialized organizations, including the American Society of Clinical Oncology (ASCO), the NCCN, and the European Society of Medical Oncology (ESMO), have published guidelines regarding VTE prophylaxis and treatment in the oncology population.

If anticoagulation is recommended, the pharmaceutical agent choice is based on the presence of renal impairment, US Food and Drug Administration approval, cost, ease of administration, need for therapeutic monitoring, availability, and ease of reversibility (NCCN, 2011). Agents that may be used based on patient characteristics include low molecular weight heparins (LMWHs), unfractionated heparin (UFH), fondaparinux, aspirin, or warfarin. The LMWH options include dalteparin (Fragmin) 5,000 units SC daily, enoxaparin 40 mg SC daily, or tinzaparin(Innohep) 4,500 units SC daily (NCCN, 2011; Lyman et al., 2007). The recommended dose of fondaparinux is 2.5 mg SC daily (NCCN, 2011; Lyman et al., 2007). Unfractionated heparin is recommended to be prescribed at 5,000 units SC three times day (NCCN, 2011; Mandala, Falanga, & Roila, 2009). Finally, aspirin and warfarin doses are individualized based on patient characteristics (NCCN, 2011).

**HOSPITALIZED CANCER PATIENTS** 

Studies have found that all hospitalized patients, including those in nononcology settings, benefit from the use of LMWH or fondaparinux for thromboprophylaxis (Samama et al., 1999; Leizorovicz et al., 2004; Cohen et al., 2006). While these data include only a small percentage of oncology patients, ASCO and NCCN agree that prophylactic anticoagulation should be considered for all hospitalized cancer patients due to their natural hypercoagulable state (Khorana, 2007; Lyman et al., 2007). However, contraindications to anticoagulation include major active bleeding, intracranial or spinal lesions at high risk for bleeding, thrombocytopenia or severe platelet dysfunction, recent surgery, spinal anesthesia or lumbar puncture, and high risk for falls (Khorana, 2007; Lyman et al., 2007). The NCCN guidelines also state that ambulation is not sufficient for VTE prevention in this population, and that hospitalized patients should wear graduated compression stockings (Khosravi-Shahi & Perez-Manga, 2009; NCCN, 2011). The ESMO guidelines state that only immobilized hospitalized cancer patients require prophylactic anticoagulation (Mandala, Falanga, & Roila, 2009).

All of the guidelines recommend the use of low-dose UFH, LMWH, or fondaparinux for prophylaxis without a particular preference (Khorana, 2007; Lyman et al., 2007; Mandala, Falanga, & Roila, 2009). However, LMWHs have been shown to have a significant impact on lowering VTE rates without a significant increase in major bleeding complications (Khorana, 2007). For patients who do have contraindications to anticoagulation, mechanical prophylaxis should consist of sequential compression devices or graduated compression stockings during hospitalization (Khorana, 2007).

**SURGICAL CANCER PATIENTS** 

The risk of VTE for the cancer patient in the postoperative period is both immediate and prolonged (Agnelli et al., 2006; Spyropoulos, 2009). Because of this, the ESMO, NCCN, and ASCO guidelines are in agreement that oncology patients in the surgical setting require anticoagulation thromboprophylaxis both initially and 7 to 10 days after surgery (Khorana et al., 2009; Mandala et al., 2009; Lyman et al., 2007; Spyropoulos, 2009). Patients that undergo major abdominal or pelvic surgery with high-risk features such as residual tumor, obesity, and history of VTE require prolonged anticoagulation for up to 4 weeks (Khorana et al., 2009; Mandala et al., 2009; Lyman et al., 2007; Spyropoulos, 2009). Both NCCN and ASCO guidelines state that mechanical prophylaxis should be used in conjunction with anticoagulation (Streiff, 2009). Ideal anticoagulation agents include LMWH and UFH as first-line agents for both hospitalization and outpatient prophylaxis (Lyman et al., 2007; Khorana et al., 2009; Spyropoulos, 2009). The use of vitamin K antagonists, such as warfarin, and aspirin are not recommended (Spyropoulos, 2009). Prophylaxis should commence either preoperatively or as early as possible postoperatively (Lyman et al., 2007)

**AMBULATORY CANCER PATIENTS** 

There are limited studies looking at the role of thromboprophylaxis in the ambulatory cancer patient. Because of this, ESMO, NCCN, and ASCO do not recommend the routine use of antithrombotic agents for VTE prophylaxis (Lyman et al., 2007; Mandala et al., 2009; Khorana et al., 2009). Studies have identified that the risk of bleeding often outweighs the risk of VTE, except for multiple myeloma patients (Streiff, 2009). This is due to the identified increased risk of VTE in patients receiving antiangiogenic agents like thalidomide and its derivatives (NCCN, 2011). The organizational guidelines are in agreement that patients receiving these agents require thromboprophylaxis. ASCO and NCCN specifically identify that LMWH or warfarin should be used in myeloma patients receiving thalidomide with chemotherapy or dexamethasone (Lyman et al., 2007; NCCN, 2011). NCCN guidelines recommend aspirin 81 mg to 235 mg for low-risk myeloma patients (NCCN, 2011). Aspirin is not recommended for nonmyeloma patients for VTE prophylaxis (NCCN, 2011). In addition, prophylaxis is currently not recommended for patients receiving hormone therapy in an ambulatory setting (Mandala et al., 2009).

**CANCER PATIENTS WITH CENTRAL VENOUS CATHETERS** 

Early studies identified that there was a benefit with low-dose warfarin use in decreasing the risk of CVC-related thrombosis (Bern et al., 1990); however, recent studies showed no benefit from anticoagulation prophylaxis (Verso et al., 2005; Couban et al., 2005; Niers et al., 2007). Because of this, the NCCN and ASCO VTE guidelines do not recommend routine prophylaxis until effective regimens have been identified (Streiff, 2009).

## Implications for the Advanced Practitioner

Despite an increasing awareness of cancer-associated VTE and the publication of evidence-based guidelines, surgical oncologists report only 52% use of prophylaxis, and medical oncologists report its use in less than 5% of their patients (Kakkar, Levine, Pinedo, Wolff, & Wong, 2003). The consequences of poor VTE prophylaxis range from deep-vein thrombosis, progression to pulmonary embolism, increased hospital-acquired conditions, deep-vein thrombosis recurrence, and death (Clarke, 2010), causing an increase in economic burden (Kessler & Cap, 2009). Because of these poor outcomes, there is an urgent need to improve compliance with nationally accepted recommendations (Clarke, 2010).

In order to improve compliance with guidelines, there has to be a change in health-care culture. The Joint Commission and Centers for Medicare and Medicaid Services are assisting in this by issuing regulations to improve the use of VTE prophylaxis (Clarke, 2010). However, it is also dependent on individual practitioners’ communication and guidance to help facilitate this paradigm shift in cultural change. As APs, it is essential to be proactive and promote the use of evidence-based guidelines similar to those developed by the NCCN, ASCO, and ESMO to improve patient outcomes. This can be done through educational initiatives, decision support tools, quality improvement with audit and feedback processes, organizational changes, and policy development (Table 4; Clarke, 2010; Khorana, 2007). There is no difference in effectiveness between the different methodologies; however, utilization and integration of multiple methodologies is best (Khorana, 2007).

**Table 4:**
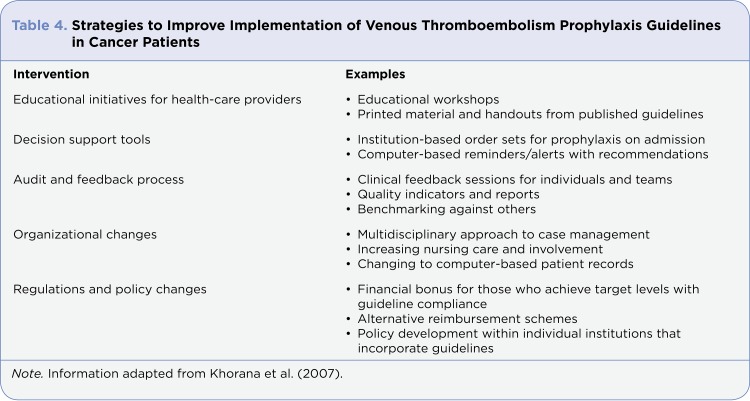
Table 4. Strategies to Improve Implementation of Venous Thromboembolism Prophylaxis Guidelines in Cancer Patients

In addition, continued research is needed, particularly in the form of large, randomized, controlled clinical trials that consist of a cancer population. Specific areas of study needed include examining the risk of toxicities associated with prophylactic anticoagulation, such as the risk of increased bleeding and quality-of- life issues in both the ambulatory and hospitalized oncology population (Khorana et al., 2009). Additional support is needed in validating appropriate prophylaxis for the oncology patient with a CVC (Khorana et al., 2009; Lyman et al., 2007). In addition, the implications that the newly developed chemotherapy-associated VTE risk assessment have on thromboprophylaxis guidelines need to be investigated thoroughly.
